# Dietary Quality of Pregnant Women Participating in the Special Supplemental Nutrition Program for Women, Infants, and Children

**DOI:** 10.3390/ijerph18168370

**Published:** 2021-08-07

**Authors:** Arezoo Rojhani, Ping Ouyang, Angel Gullon-Rivera, Taylor Marie Dale

**Affiliations:** Department of Family and Consumer Sciences, Western Michigan University, Kalamazoo, MI 49008-5322, USA; ping.ouyang@wmich.edu (P.O.); angel.gullon-rivera@wmich.edu (A.G.-R.); taylor.dale@wmich.edu (T.M.D.)

**Keywords:** dietary quality, pregnant women, HEI-2015, Special Supplemental Nutrition Program for Women, Infants, and Children, WIC, HEI score, nutrient analysis, micronutrients

## Abstract

Few studies have examined the dietary intake of low-income pregnant women participating in the Special Supplemental Nutrition Program for Women, Infants, and Children (WIC). The aim of this study was to assess the dietary quality of WIC-enrolled pregnant women and examine associations with maternal characteristics, nutrition knowledge, and key health indicators. Fifty-one WIC-enrolled pregnant women completed two sets of 3-day food records. Food records were analyzed for nutrient content, and diet quality was assessed using the Healthy Eating Index (HEI)-2015. Since an HEI score of less than 60 is indicative of the need to improve dietary quality, participants’ HEI scores were divided into two categories: <60 and ≥60. The total mean HEI score of the cohort based on analysis of the first set of food records was 59.1 ± 12.5 (range 37.1–89.2), while the mean score for the second 3 days of food records was 56.8 ± 12.7 (range 30.0–89.0). The majority of participants did not consume the minimum recommended servings of whole vegetables. Those in the <60 HEI category consumed on average less than 50% of the recommended servings of whole fruits and whole grains. The diets of the majority of participants were high in saturated fat and sodium. More than one-third did not meet the recommendations for folate and iron intake, while less than half met the RDA for vitamin D. Choline intake was insufficient based on analysis of the first 3 days of food records. Our results indicate that the dietary quality of WIC-enrolled pregnant women requires improvement.

## 1. Introduction

Pregnancy is a critical phase in a woman’s life, when dietary practices can not only have a profound effect on the health of both the mother and the fetus, but can affect the future health of the offspring [1,2]. Studies have shown that the metabolic and hormonal conditions of the intrauterine environment that are influenced by diet and physical activity play an important role as epigenetic factors affecting health and risk of disease in later life [3,4,5,6]. These include effects on bone mass, risk of obesity and diabetes during adolescence and adulthood, and future mental health, among others [7,8,9,10].

Despite the proven relationship between maternal nutrition and pregnancy outcomes, the diet of many pregnant women does not align with dietary recommendations [11,12]. To address this problem, many countries have developed specific dietary guidance for pregnant women [13,14]. Results of a Canadian study assessing the dietary quality of pregnant women reported that 40% of participants did not meet the minimum recommended number of servings of the Food Guide Pyramid, and only 4% met folate recommendation, while none met iron recommendations [11]. Data from studies carried out in the United States (US) have shown similar patterns, with an inadequate intake of key micronutrients and overall low quality of diet for pregnant women [1,2,3,4,5,6,7,8,9,10,11,12,13,14,15,16]. Research from Greece has demonstrated significantly lower intakes of folate and iron compared to the Dietary Reference Intakes (DRIs), and a mediocre dietary quality for a sample of pregnant women living in Athens; in this study, women who were underweight or of normal body weight had better diet quality than obese women [17]. Results of a Singaporean study carried out to validate a dietary quality assessment tool (HEI-SGP) for pregnant women reported a mean HEI-SGP score of 52.4 out of 100 points [18].

Adhering to a diet that supplies an adequate amount of energy and balance of nutrients that are important during pregnancy is especially challenging for low-income pregnant women. Studies have shown inadequate dietary quality and low intakes of iron, folate, and fruits and vegetables among low-income pregnant women [19,20]. To address this public health concern, some countries have put safety nets in place. In the US, the Special Supplemental Nutrition Program for Women, Infants, and Children (WIC), funded by the US government, was established in 1972 to safeguard the health of low-income pregnant women, breastfeeding and non-breastfeeding postpartum women, and infants and children up to the age of five, through the provision of supplemental foods, health care referrals, and nutritional education [21]. Four in ten women (39.6%) who gave birth in the US in 2016 received prenatal services from the WIC [22]. Although a number of studies have reported on the positive effects of the WIC program on birth outcomes [23,24,25], very few have assessed the dietary quality of pregnant women enrolled in the WIC program [26,27]. The aim of this study was to evaluate the dietary quality of pregnant women participating in the WIC program, and to examine possible associations with demographic variables, maternal characteristics, nutritional knowledge, and key health indicators.

## 2. Materials and Methods

### 2.1. Sample Recruitment

WIC-enrolled pregnant women who were between 12 and 24 weeks of gestation and were carrying a single fetus were recruited from two WIC clinics in Kalamazoo, Michigan. The contact information of pregnant women who met the inclusion criteria was provided to the researchers by the WIC clinics on a weekly basis. A total of 500 pregnant women who met the eligibility criteria were invited; of these, 60 consented to participate in the study. Participants were asked to complete a survey instrument that consisted of four domains and included open- and closed-ended questions. The first two domains included questions on the socio-demographic and maternal characteristics, key health indicators, as well as questions assessing knowledge of anemia, iron-deficiency anemia, anemia during pregnancy, and food sources of iron. Information on the development of the survey instrument and the process used to establish validity has been previously published [28]. Pre-pregnancy BMI was calculated using self-reported height and weight. The pre-pregnancy and current weight of the women and their gestational age were used to calculate the adequacy of maternal weight gain during pregnancy based on the Institute of Medicine 2009 Gestational Weight Gain Guidelines [29]. Study participants’ blood hemoglobin concentrations were obtained from their WIC records.

### 2.2. Dietary Assessments

Participants were asked to complete two sets of 3-day food diaries. The first set was completed soon after joining the study, while the second set was completed around 28 weeks of gestation. All participants scheduled an in-person interview with the registered dietitian, who was a research assistant for the project, and received training using food models and household measuring utensils on how to accurately estimate serving sizes before completing the first set of 3-day food diaries. After completion of each set of food diaries, the research assistant reviewed the food diaries in the presence of the participants to ensure accuracy. Participants received gift cards to a large grocery store with multiple locations in Kalamazoo for completing each phase of the project. Although all 60 participants completed the survey instrument, only 51 submitted two complete sets of 3-day food diaries. All of the food diaries were analyzed for nutrient content using the Food Processor Nutrition Analysis Software (version 11.7 ESHA Research, Salem, MA, USA). The quality of the diet of each participant was assessed using the Healthy Eating Index (HEI)-2015 twice—once based on the first set of 3-day food diaries, and again around 28 weeks of gestation using the second set of 3-day food records. The HEI was developed by the United States Department of Agriculture (USDA), and is a food-based index that provides an assessment of the degree to which an individual’s diet aligns with the recommendations of the Dietary Guidelines for Americans (DGA). HEI-2015, the latest version of this diet-quality index, has been shown to be a validated measure of dietary quality, and is comprised of 13 components [30,31]: the first 9 components emphasize dietary adequacy, meaning that a person should consume at least the given equivalent amounts; the other 4 components emphasize moderation, meaning that a person should eat less than the given equivalent amounts. Each of the dietary components has a maximum score of 5 or 10 points, with the total points for all 13 categories equaling 100. To account for differences in caloric intake, the score is found by calculating the amounts consumed per 1000 calories. For each component, participants with an intake at the recommended level received a perfect score of 5 or 10, while a score of 0 was assigned if no food from a component was consumed. Intermediate intakes were scored and calculated proportionately. To assess dietary quality, an HEI score of 80 or above out of a total of 100 points was used as the cutoff to denote a “good-quality” diet, while a score of less than 60 wasindicative of “need to improve” [31]. Since very few of the cohort in this study had HEI scores of 80 or above, rather than creating tertiles of HEI scores—the approach used by other investigators [17,18]—two categories of HEI scores were created: <60 and ≥60. Intakes of macro- and micronutrients and energy were compared to the Institute of Medicine’s Dietary Reference Intakes (DRIs) during pregnancy [32]. This study was approved by the Institutional Review Board of Western Michigan University (protocol code 15-07-03; approved on 13 August 2020).

### 2.3. Statistical Analysis

Descriptive statistics were used to evaluate maternal characteristics, HEI scores, energy, and nutrient intake. Chi-squared tests were used to determine differences in maternal characteristics between the two categories of HEI (<60 and ≥60) for the first and second sets of 3-day food records when variables were categorical or normally distributed. Comparisons between the first and the second sets of 3-day food records for total HEI scores, HEI component scores, and nutrient intakes were carried out using repeated-measures analysis of variance (ANOVA), with demographic characteristics (age, marital status, education, working status, and ethnicity), pre-pregnancy BMI, smoking, morning sickness, and additional food assistance programs as between-group factors. Greenhouse–Geisser corrections were applied wherever the sphericity assumption was not met. The independent samples *t*-test was used to test for differences between the means for each of the 13 HEI components, comparing the HEI < 60 and HEI ≥ 60 categories. Since the nutrient intake data were not normally distributed within each group of HEI categories (<60 and >60), the Mann–Whitney U test was used to compare differences in energy intake and % Dietary Reference Intake (DRI) of nutrients between the two categories of HEI (<60 and ≥60) for each set of 3-day food records. Pearson’s correlation analysis was conducted to examine the relationships between the HEI scores and demographic variables, maternal characteristics, key health indicators, and nutritional knowledge. Hierarchical regression analyses were computed separately with both sets of 3-day records to examine potentially significant demographic and maternal characteristic factors associated with HEI scores. All analyses were completed using the Statistical Package for Social Sciences (SPSS), version 27. In all analyses, a *p*-value of ≤0.05 was used to designate statistical significance.

## 3. Results and Discussion

The overview of the maternal characteristics of study participants is presented in Table 1. The majority were non-Hispanic white, with more than one-third non-Hispanic black. Few of the participants identified as Hispanic, Asian, or other races.

More than three-quarters of our sample had obtained a college degree. Most did not smoke prior to pregnancy. Of those who smoked, two-thirds quit smoking once aware of the pregnancy. The great majority reported using prenatal supplements. Among non-users, reasons given included extreme nausea and forgetfulness. The majority of the study cohort was only receiving food assistance from the WIC, which indicates that their income was ≤185% of the federal poverty level (the family income eligibility criterion for receiving WIC) Based on chi-squared analysis, no statistically significant differences in maternal characteristics were found between participants in the <60 and ≥60 HEI categories for the first or thesecond survey.

Table 2 shows the HEI component scores and the percentage of recommended serving equivalents for each component, as well as the overall mean HEI scores for the first 3 days of food records (survey 1) and the second set of 3-day food records (survey 2). ‘The overall score of the cohort based on analysis of the first 3 days of food records was 59.1 ± 12.5 (range 37.1–89.2), while the mean score for the second 3 days of food records was 56.8 ± 12.7 (range 30.0–89.0). These scores are 4–6 points lower than the mean HEI score of 63 reported by the USDA for pregnant women who participated in the National Health and Nutrition Examination Survey (NHANES) in 2013–2016 [33]. However, the overall dietary quality scores of our participants were similar to what was reported by the only other two studies we found that evaluated the dietary quality of this population [26,27]. An HEI score of 80 or above out of a total of 100 points is an indication of a “good-quality” diet [13,30,31]. In our population of pregnant mothers, only four (7.8%) had an HEI score of ≥ 80 based on evaluation of the first 3 days of food records, while based on analysis of the second set of 3-day food records, only two (3.9%) of the participants were in the ≥ 80 HEI category.

Since the HEI is density-based (i.e., amounts consumed per 1000 kcal), to interpret scores—especially low scores—it is important to examine energy intake and evaluate scores for each component of the HEI [31]. Based on the mean of the data for the HEI components presented in Table 2, except for total protein foods and added sugars, participants did not meet the recommendations for any of the other 11 components during survey 1 and survey 2. This was especially noticeable for the total vegetables, fatty acids, sodium, and saturated fat, where the mean scores decreased from survey 1 to survey 2. However, the difference in mean scores between survey 1 and survey 2 was only significant for saturated fats (*p* < 0.003). Since the fatty acids equivalent is the ratio of monounsaturated fatty acids (MUFAs) plus polyunsaturated fatty acids (PUFAs) to saturated fatty acids (SFAs), these low percentages plus the low HEI component scores for saturated fats indicate that the participants had low intakes of MUFAs and PUFAs and higher intake of SFAs. The high intake of saturated fats of our study participants is concerning, as this can increase their risk for cardiovascular disease [34,35].

Since an HEI score of less than 60 is indicative of the need to improve dietary quality, we divided the participants into two categories (<60 and ≥60) to determine which of the HEI components were consumed and which ones were not consumed at the recommended level. Table 3 shows the mean HEI component scores and the percentage of the recommended serving equivalents met for each component, for survey 1 and survey 2, by HEI category. Overall, the pregnant women in the ≥60 HEI category met the recommended intake for each of the 13 HEI components more closely than those in the <60 HEI category. The mean percentage of recommendations met was especially low for the total vegetables and fatty acids components for both HEI categories. Compared to those in the ≥60 HEI category, participants in the <60 HEI category had significantly lower intakes of total fruits, whole fruits, whole grains, and dairy, and higher intakes of sodium and saturated fats, in both surveys 1 and 2. The differences in HEI component scores for seafood and plant proteins for the two categories of HEI were not statistically significant in survey 1 or 2, but were trending towards significance. The total mean HEI score of participants in the ≥60 HEI category was significantly higher than that of those in the <60 category (*p* < 0.001) for both surveys 1 and 2.

Evaluation of the second 3 days of food records demonstrated that despite the slight improvement in the total HEI scores of participants for both the <60 and ≥60 HEI categories, the gap in the mean scores for some HEI components widened (Table 3). For example, while participants in the ≥60 HEI category had an 11.4 % increase in their total vegetable intake, those in the <60 HEI category experienced a 9.0% decline. Our data on low intakes of vegetables and whole grains by our pregnant mothers are consistent with what has been previously reported for this population [27], and for low-income pregnant women as a whole [20].

Hamad et al. [26] reported a 2.4-point increase in the total HEI score and a slight improvement in the fruit and fat intake of pregnant women participating in the WIC program as a result of revisions to the WIC program food package that went into effect in 2009. The revised food package provides more fruits, vegetables, and whole grains, and restricts milk purchases to low-fat milk. The authors commented that although these improvements were minor at the individual level, the impact at the population level would be much greater. Despite the improvements noted by Hamad et al., based on the data from the present study, there is clearly a need for further improvement in the dietary quality of WIC-enrolled pregnant women.

Table 4 shows a comparison of calorie and nutrient intakes for the first 3 days of food records (survey 1) and the second set of food records (survey 2). There were significant increases in the percentage of calories derived from total fat, MUFAs, and PUFAs, and a decrease in the percentage of calories derived from added sugars. There were also significant increases in the intake of fiber and several of the micronutrients of importance for pregnant women, including vitamin D, iron, folate, choline, and zinc. It may seem as though our participants were able to meet the nutritional needs of pregnancy more closely based on the survey 2 food records; however, this needs to be evaluated in the context of their energy intake. The overall mean energy intake of our participants during the second survey was 2613 kcal (Table 4), or 98.6% of the upper limit of recommended dietary allowance (RDA) for energy during the second and third trimesters of pregnancy. Forty-seven percent of our sample consumed in excess of the RDA for energy.

When one consumes more calories, naturally, the intake of many nutrients will increase; however, this does not mean that dietary quality has improved. The latter is supported by the slight decrease in the overall meanHEI scores of participants in survey 2 compared to survey 1 (Table 2).

The mean pre-pregnancy BMI for our cohort was 30.1 ± 8.75 (Table 1). Thirty-one percent of our participants were overweight, while 39.2% were obese prior to pregnancy. A high prevalence of overweight and obesity has also been reported among WIC-enrolled pregnant women from Minnesota [36] and Michigan [33]. Furthermore, 47% of the pregnant women in this study gained more than the recommended amount of weight based on their gestational age. Obesity prior to pregnancy and excess gestational weight gain are thought to not only result in pregnancy complications and poor health for the expectant mother, but have also been shown to independently exert long-term health effects on the developing child, including higher body fat levels in infants, obesity in children, insulin resistance, elevated blood lipid levels, and hypertension [3,6]. Studies have shown that pregnant women are more willing to make lifestyle changes for the sake of their unborn child; this, combined with the fact that they are seen more regularly by their health care providers, provides a window of opportunity for lifestyle interventions [6].

Table 5 shows a comparison of the calorie and nutrient intakes of participants in the lower (<60) and higher (≥60) HEI categories for surveys 1 and 2. Nutrient analysis of the first 3 days of food records showed similarity in the macronutrient composition of diets between the <60 and ≥60 HEI categories. However, the mean energy intake for the participants in the <60 HEI category was approximately 300 kcal higher than those in the ≥60 HEI category, but the difference was not statistically significant (Table 5). The percentage of energy derived from total fat was at the high end of the recommended intake of 20–35% of calories from fat [35] for both HEI categories. Those in the <60 HEI category narrowly missed exceeding the total fat intake recommendations. The mean percentage of saturated fat intake of participants in both HEI categories (<60 and ≥60) exceeded the 2015–2020 DGA recommendation of consuming less than 10% of calories from saturated fat [35]. Pregnant women in both HEI categories complied with the 2015–2020 DGA recommendation of added sugars not exceeding 10% of overall calorie intake per day [35]. Nearly 22% of participants in the ≥60 HEI category, and 35% in the <60 category, fell short of the 28 g/day recommendation of fiber intake for pregnant women [35]. The difference in fiber intake between the two categories of HEI was statistically significant (*p* = 0.05).

Micronutrient analysis of the diet using the first set of food records revealed excessively high intake of sodium, with a statistically significant difference between the <60 and ≥60 HEI categories (*p* < 0.03), and a fairly low vitamin D intake for both HEI categories (Table 5). Vitamin D deficiency is common in pregnancy, with a reported prevalence as high as 50% [37]. The latter is partly because there are only a handful of good dietary sources of this fat-soluble vitamin. These include fatty fish, eggs, fortified milk, yogurt, and fortified cereals and juices [38]. Intake of calcium for both HEI categories was reasonably adequate, with those in the <60 category falling slightly short of the RDA for calcium. A large percentage (96%) of those in the ≥60 HEI category, and about 81% of those in the <60 HEI category, met the RDA for zinc.

While the vitamin B_12_ intake of our sample exceeded the RDA for pregnant women, intakes of folate, iron, and choline were lower than the recommendations (Table 5). This was especially pronounced for folate and iron in the <60 HEI category, with 48% and 44% falling short of the RDAs for folate and iron, respectively. The differences in iron, folate, and choline intake between the two HEI categories were not statistically significant. Inadequate intake of iron and folate by our cohort is consistent with the results of two other studies that examined dietary intake of WIC-enrolled pregnant women and reported similar findings [27,36].

To date, studies that have evaluated the dietary adequacy of WIC-enrolled pregnant women have not examined their choline intake. Choline is an important micronutrient during pregnancy, as it plays an important role in placental function, neurodevelopment (processing speed, visuospatial memory, attention, self-regulation, and visual acuity), and epigenetic programming (neonatal stress reactivity, fetal growth, brain development, and chronic disease risk) [38]. Based on NHANES data, the percentage of US pregnant women who reached an adequate intake level of this nutrient was less than 10% [39]. In 2018, choline was termed the “brain-building” nutrient by the American Academy of Pediatrics, which called upon pediatricians to ensure that pregnant women and young children have adequate intakes of choline. Recommendations regarding choline intake have been added to the 2020 US Dietary Guidelines for Pregnant and Lactating Women [40]. Since choline is not included in most prenatal vitamin supplements, some have proposed that women with low intakes of choline-rich foods (e.g., meat, fish, eggs, milk) consider taking a dietary supplement containing this micronutrient.

There were no significant differences between the two categories of HEI scores with respect to the mean energy and macronutrient intake based on analysis of the second set of food records (Table 5). However, the percentage of calories derived from carbohydrates was marginally adequate for both HEI categories, while the percentage of calories from total fat for the <60 HEI category exceeded the recommendation of the DGA. Those in the ≥60 HEI category met the total fat intake recommendations marginally. Both HEI categories exceeded the DGA recommendation for saturated fat intake. Swensen et al. [36] reported a similar dietary pattern for their sample of pregnant WIC recipients, consisting of a high intake of fat (37% of calories) and inadequate intake of carbohydrates. Adoption of a high-fat diet during pregnancy has also been reported for other populations of pregnant women [17]. The mean percentage of AI for fiber was significantly higher for the ≥60 HEI category compared with the <60 category, with 88% of the recommendation for fiber intake during pregnancy met (*p* = *0*.009).

The results of micronutrient intake analysis of the second set of food records showed a similar pattern for sodium as to what was described for the first set of food records, with the mean percentage of sodium intake for both HEI categories much higher than the AI for sodium. The mean for the <60 category was more than three times higher than the sodium AI (Table 5). The pattern with vitamin D intake was also similar to what was observed based on analysis of the first set of food records, with only about one-third of those in the <60 category, and slightly more than 50% of those in the ≥60 HEI category, meeting the requirements. Both HEI categories fell short of the recommended intakes of folate and iron during pregnancy, with the difference between the lower and higher HEI categories trending toward significance for iron (*p* = 0.08). The mean % RDAs for vitamin B_12_, calcium, and zinc were more than adequate for both HEI categories, with the differences between the lower and higher HEI categories statistically significant for vitamin B_12_ (*p* = 0.04) and zinc (*p* = 0.04). The mean intake of choline was slightly lower than the AI for the <60 HEI category compared to a significantly lower mean intake for the ≥60 HEI category (*p* < 0.04).

Using an older version of the HEI (HEI-2005) to compare the dietary quality of young pregnant and non-pregnant women, Pick et al. [11] concluded in their samples of pregnant women with a mean HEI score of 75 but diets severely deficient in folate and iron that the HEI was not sensitive enough to pick up micronutrient deficiencies. In this study, the mean % RDA or AI met for the majority of micronutrients evaluated was higher (reaching statistical significance for some) for those in the ≥60 HEI category compared to those in the <60 category. To investigate this further, we evaluated the micronutrient intakes of the six participants who had overall HEI scores of 80 or above—the hallmark of a “good-quality” diet. Analysis of data showed that with the exception of vitamin D—where only 32% met the RDA—and choline—with 60.7% meeting the AI—the requirements for all other micronutrients (vitamin B_12,_ folate, iron, and calcium) were satisfied through diet alone. Of course, both studies consisted of small sample sizes; therefore, the suitability of the HEI for the evaluation of the dietary quality of pregnant women requires additional investigation.

The distribution of participants between the two HEI score categories (<60 and ≥60) for surveys 1 and 2 is shown in Table 6. Slightly more than half of the participants were in the <60 HEI score category based on analysis of the first 3 days of food records, while two-thirds were in the low-HEI category based on analysis of the second set of food records. The difference in the distribution of participants between the <60 and ≥60 HEI categories was statistically significant for the second set of food records (*p* ≤ 0.05). This is concerning, as it indicates that the overall quality of the diet of some of the pregnant women in our study deteriorated as their pregnancy progressed. These results are consistent with what was reported by Moran et al. for Australian pregnant women [10]; in this study, the authors reported a decrease in the dietary quality of their overweight and obese pregnant women as the gestational period progressed. Similarly, Tsigga et al. [17] reported a significant negative correlation between dietary quality and weeks of gestation in their sample of pregnant women from Greece. Given the high prevalence of overweight and obesity in our sample, it is possible that some of these pregnant women succumbed to temptations as their pregnancy progressed, resulting in poor dietary quality.

Correlational analysis indicated several significant relationships between the HEI scores, nutritional knowledge, pre-pregnancy BMI, and blood hemoglobin concentrations of participants (Table 7). The HEI scores based on both survey 1 (r = 0.41, *p* = 0.003) and survey 2 (r = 0.32, *p* = 0.024) were positively correlated with the knowledge of anemia score. In addition, the HEI scores obtained from the analysis of the survey 1 food records were negatively correlated with the pre-pregnancy BMI of the study cohort (r = −0.31, *p* < 0.05). Pregnant women who had higher BMI had lower HEI scores. The latter has been reported by other researchers who have evaluated the association between diet quality and pre-gravid and gravid weight status [14,17]. There was also a positive correlation between the blood hemoglobin concentration of participants and their HEI scores obtained from the analysis of the survey 2 food records (r = 0.44, *p* < 0.01); those with higher HEI scores were less likely to be anemic.

Hierarchical regression analyses were performed with both sets of 3-day food records in order to examine the factors related to the HEI (Table 8 and Table 9). The results from the first set were similar to those from the second set; neither analysis revealed multicollinearity. The tolerance and variance inflation factors (VIFs) were within the acceptable range among predictors. The tolerance ranged between 0.796 and 0.819, while the VIF values obtained were between 1 and 10 (ranging from 1.22 to 1.73). The condition index was less than 15; thus, multicollinearity was not a concern. The goal of the regression analysis was to ascertain the best model to explain the variance in the HEI scores. In the first step, education, working status, marital status, age, and ethnicity were considered; in the second step, smoking, morning sickness, pre-pregnancy BMI, food assistance programs, and blood hemoglobin concentration were added; in the final step, knowledge of anemia was entered (Table 8). The final step examined the incremental changes in HEI scores as knowledge of anemia was separately entered.

We found that demographic variables entered at the first step were not significant. After adding smoking, morning sickness, pre-pregnancy BMI, food assistance programs, and blood hemoglobin concentration, the demographic variables remained non-significant factors. The addition of knowledge of anemia as another potential factor in the final step was significant for both sets of 3-day food records. In the analysis of the second set of 3-day food records (survey 2), blood hemoglobin concentration was significant in the final step, accounting for 8% of the variance in HEI scores. This supports the data from the correlational analysis, where a positive and significant association was found between blood hemoglobin concentrations and the HEI scores of participants. Including knowledge of anemia in the final model as a whole explains 25% and 35% of the variance in HEI scores for survey 1 (*p* < 0.019) and survey 2 (*p* < 0.040), respectively—that is, participants who had higher HEI scores had more nutritional knowledge.

Based on our data, very few of the study participants were able to follow the 2015–2020 recommendations of the DGA closely. The latter is reflected in their low total HEI and HEI component scores. The majority of the participants did not consume the minimum recommended number of servings from the total vegetables component. Those in the low-HEI category, on average, consumed only ~50% of the minimum recommended servings for whole fruits. In addition, most consumed too much saturated fat, and not enough MUFAs and PUFAs. Participants in the low-HEI category consumed less than 50% of the recommended servings for whole grains. However, most participants complied with the added sugar recommendations. Analysis of micronutrient intake revealed that regardless of the HEI category, the study participants consumed well above the AI for sodium. More than one-third did not meet the recommendations for folate and iron, while less than half met the RDA for vitamin D. Although some women clearly did not consume enough choline, overall, intakes improved as pregnancy progressed, reaching more than 90% of the AI for women in the low-HEI category based on analysis of the second set of 3-day food records. Based on chi-squared analysis, the quality of the diets of some participants deteriorated as they began their third trimester. Given the gaps in dietary quality discussed above, using prenatal vitamin and mineral supplements that provide adequate amounts of all required micronutrients is critical for this population.

Limitations of this study include its cross-sectional design and relatively small sample size, which was gathered by convenience. In addition, most of our participants were non-Hispanic white or non-Hispanic black. A review of the National Center for Health Statistics (NCHS) data brief [22] indicates that the majority of pregnant WIC-eligible recipients are Hispanic. We had very few Hispanics in our sample; therefore, a future study, which better represents the racial diversity of the US WIC-eligible pregnant population utilizing a larger sample size, is recommended. Given that this was a pilot study with a small sample size, its findings cannot be generalized. However, this is the first study that has used HEI-2015 to assess the dietary quality of WIC-enrolled pregnant women, and the first to provide a detailed analysis of the micronutrient intake of this population.

## 4. Conclusions

This study provides a comprehensive evaluation of the diets of pregnant women participating in the WIC program. Our data indicate that the dietary quality of WIC-enrolled pregnant women requires improvement. Given that the influence of maternal diet goes beyond the mother’s own health, and can affect the future health of her offspring, provision of more nutritional education that targets the specific needs of pregnancy may be beneficial to this population.

## Figures and Tables

**Table 1:** Maternal characteristics of study participants (*n* = 51).

Characteristics		*N* (%)	Mean (SD)
Age, years			27.88 (5.74)
Pre-pregnancy BMI			30.10 (8.75)
Race			
	White	24 (47.1%)	
	Black	19 (37.3%)	
	Hispanic	3 (5.9%)	
	Asian	1 (2.0%)	
	Other race	4 (7.8%)	
Ethnicity			
	Hispanic	3 (5.9%)	
	Non-Hispanic	48 (94.1%)	
Marital status			
	Single	20 (39.2%)	
	Single with live-in companion	13 (25.5%)	
	Married	18 (35.3%)	
Work status			
	Full-time	9 (17.6%)	
	Part-time	13 (25.5%)	
	Part-time + School	1 (2.0%)	
	School only	4 (7.8%)	
	Not working or school	24 (47.1%)	
Education level			
	Less than high school	5 (9.8%)	
	High school	6 (11.8%)	
	College degree	40 (78.4%)	
Smoking			
	No	33 (64.7%)	
	Yes	18 (35.3%)	
Prenatal supplement use			
	Yes	43 (84.3%)	
	No	8 (15.7%)	
Food assistance program *			
	WIC ** only	32 (62.7%)	
	WIC + SNAP ***	18 (35.3%)	

Notes: * missing response *n* = 1; ** WIC—Special Supplemental Nutrition Program for Women, Infants, and Children; *** SNAP—Supplemental Nutrition Assistance Program.

**Table 2:** Comparison of HEI scores of participants for survey 1 and survey 2.

HEI Components	Survey 1		Survey 2		*p*-Value *
	Mean (±SD)	% Recommendation Met	Mean (±SD)	% Recommendation Met	
Total fruits ^A^	3.23 (±1.94)	64.6	3.27 (±1.92)	65.4	0.904
Whole fruits ^A^	3.39 (±2.09)	67.8	3.07 (±2.07)	61.4	0.347
Total vegetables ^A^	2.09 (±1.82)	41.8	1.92 (±1.73)	38.4	0.637
Greens and beans ^A^	2.83 (±2.26)	56.6	2.80 (±2.31)	56.0	0.941
Whole grains ^B^	5.33 (±4.10)	53.3	5.42 (±4.06)	54.2	0.906
Dairy ^B^	5.78 (±3.37)	57.8	6.58 (±3.21)	65.8	0.085
Total protein foods ^A^	4.78 (±0.68)	95.6	4.75 (±1.18)	95.0	0.841
Seafood and plant proteins ^A^	2.67 (±2.14)	53.4	2.08 (±2.24)	41.6	0.136
Fatty acids ^B^	2.60 (±3.08)	26.0	1.79 (±3.00)	17.9	0.073
Refined grains ^B^	6.42 (±4.08)	64.2	7.15 (±4.05)	71.5	0.141
Sodium ^B^	4.47 (±3.61)	44.7	3.66 (±3.67)	36.6	0.179
Added sugars ^B^	8.92 (±2.45)	89.2	9.31 (±1.89)	93.1	0.336
Saturated fats ^B^	6.62 (±2.55)	66.2	5.05 (±3.36)	50.5	0.003
Mean overall HEI score ^C^	59.14 (±12.56)		56.84 (±12.70)		0.220

HEI—Healthy Eating Index; ^A^ = 5 total points possible; ^B^ = 10 total points possible; ^C^ = 100 total points possible; SD—standard deviation; * *p*-values were obtained by repeated-measures ANOVA, and were considered statistically significant if ≤0.05. Survey 1—HEI < 60: *n* = 26; HEI ≥ 60: *n* = 25. Survey 2—HEI < 60: *n* = 34; HEI ≥ 60: *n* = 17.

**Table 3:** Mean HEI scores of participants in the two HEI categories for survey 1 and survey 2.

HEI Components	Survey 1			Survey 2		
	Mean (±SD)	% Meeting	*p*-Value *	Mean (±SD)	% Meeting	*p*-Value *
		Recommendation			Recommendation	
Total fruits ^A^						
HEI < 60	2.40 (±1.94)	48.0	0.001	2.75 (±1.95)	55.0	0.005
HEI ≥ 60	4.10 (±1.54)	82.0		4.31 (±1.41)	86.2	
Whole fruits ^A^						
HEI < 60	2.68 (±2.23)	53.6	0.012	2.58 (±2.08)	51.6	0.015
HEI ≥ 60	4.12 (±1.67)	82.4		4.06 (±1.71)	81.2	
Total vegetables ^A^						
HEI < 60	1.92 (±1.87)	38.4	0.512	1.47 (±1.47)	29.4	0.007
HEI ≥ 60	2.26 (±1.79)	45.2		2.83 (±1.88)	56.6	
Greens and beans ^A^						
HEI < 60	2.67 (±2.34)	53.4	0.597	2.50 (±2.29)	50.0	0.188
HEI ≥ 60	3.00 (±2.21)	60.0		3.40 (±2.28)	68.0	
Whole grains ^B^						
HEI < 60	2.89 (±3.08)	28.9	<0.001	4.16 (±3.99)	41.6	0.001
HEI ≥ 60	7.87 (±3.46)	78.7		7.93 (±2.94)	79.3	
Dairy ^B^						
HEI < 60	4.37 (±3.34)	43.7	0.001	5.51 (±3.16)	55.1	< 0.001
HEI ≥ 60	7.26 (±2.74)	72.6		8.73 (±2.08)	87.3	
Total protein foods ^A^						
HEI < 60	4.74 (±0.69)	94.8	0.71	4.75 (±1.34)	95.0	0.953
HEI ≥ 60	4.81 (±0.68)	96.2		4.73 (±0.78)	94.6	
Seafood and plant proteins ^A^						
HEI < 60	2.13 (±2.20)	42.6	0.065	1.68 (±2.09)	33.6	0.057
HEI ≥ 60	3.23 (±1.97)	64.6		2.86 (±2.39)	57.2	
Fatty acids ^B^						
HEI < 60	1.83 (±2.99)	18.3	0.067	1.20 (±2.38)	12.0	0.046
HEI ≥ 60	3.41 (±3.01)	34.1		2.96 (±3.77)	29.6	
Refined grains ^B^						
HEI < 60	6.11 (±4.38)	61.1	0.578	6.81 (±4.29)	68.1	0.396
HEI ≥ 60	6.75 (±3.81)	67.5		7.84 (±3.52)	78.4	
Sodium ^B^						
HEI < 60	3.04 (±3.16)	30.4	0.003	2.49 (±3.19)	24.9	0.001
HEI ≥ 60	5.95 (±3.50)	59.5		6.00 (±3.53)	60.0	
Added sugars ^B^						
HEI < 60	8.37 (±3.11)	83.7	0.103	9.33 (±2.10)	93.3	0.918
HEI ≥ 60	9.49 (±1.31)	94.9		9.27 (±1.44)	92.7	
Saturated fats ^B^						
HEI < 60	5.56 (±2.78)	55.6	0.002	4.36 (±3.39)	43.6	0.037
HEI ≥ 60	7.73 (±1.73)	77.3		6.42 (±2.92)	64.2	
Mean HEI Score ^C^						
HEI < 60	48.70 (±5.41)		<0.001	49.58 (±7.17)		<0.001
HEI ≥ 60	69.99 (±7.55)			71.35 (±7.92)		

HEI—Healthy Eating Index; ^A^ = 5 total points possible; ^B^ = 10 total points possible; ^C^ = 100 total points possible; SD—standard deviation; * *p*-values were obtained by *t*-test for independent samples, and were considered statistically significant if ≤0.05. Survey 1—HEI < 60: *n* = 26; HEI ≥ 60: *n* = 25. Survey 2—HEI < 60: *n* = 34; HEI ≥ 60: *n* = 17.

**Table 4:** Comparison of the nutrient intake of participants for survey 1 and survey 2.

Nutrient Intakes *	Survey 1	Survey 2	*p*-Value **
	Mean (±SD)	Mean (±SD)	
Mean total energy intake (kcal)	2389.45 (±865.81)	2612.97 (±1118.27)	0.071
Dietary protein (% of total energy)	15.89 (±4.61)	17.76 (±4.49)	0.085
Dietary carbohydrates (% of total energy)	50.92 (±8.57)	47.17 (±9.45)	0.090
* Dietary total fat (% of total energy)	33.76 (±6.58)	35.81 (±7.54)	0.050
Dietary saturated fatty acids (% of total energy)	10.71 (±2.60)	12.09 (±2.91)	0.120
^†^ Dietary monounsaturated fatty acids (% of total energy)	7.77 (±3.23)	8.38 (±3.33)	0.020
^†^ Dietary polyunsaturated fatty acids (% of total energy)	4.54 (±2.32)	4.90 (±4.60)	0.006
* Dietary added sugars (% of total energy)	4.79 (±5.85)	3.69 (±3.39)	0.029
% ^†^ AI total fiber	71.61 (±26.41)	75.83 (±40.48)	0.010
% AI sodium	274.24 (±109.15)	317.21 (±157.38)	0.092
% ^†^ RDA vitamin D	36.77 (±33.34)	42.63 (±35.35)	0.019
% RDA vitamin B_12_	206.28 (±132.38)	266.05 (±212.07)	0.073
% ^†^ RDA folate	60.02 (±33.96)	61.58 (±36.64)	0.001
% RDA calcium	100.22 (±56.14)	118.34 (±67.76)	0.104
% ^†^ RDA iron	64.92 (±38.15)	68.08 (±32.46)	0.019
% AI choline	69.02 (±42.21)	84.38 (±54.34)	0.056
% ^†^ RDA zinc	88.59 (±49.39)	108.33 (±82.20)	0.050

* Intake from dietary sources; excludes intake from supplements; ** *p*-values were obtained by repeated-measures ANOVA, and were considered statistically significant if ≤0.05. ^†^ Differences between survey 1 and survey 2 were statistically significant; HEI—Healthy Eating Index; AI—adequate intake; RDA—recommended dietary allowance.

**Table 5:** Percentage of recommended nutrient intakes of participants in the two HEI categories for survey 1 and survey 2.

Nutrient Intakes *	Survey 1		Survey 2	
	Mean (±SD)	*p*-Value **	Mean (±SD)	*p*-Value **
Mean total energy intake (kcal)				
HEI < 60	2538.19 (±967.05)	0.56	2624.00 (±1220.86)	0.8
HEI ≥ 60	2234.77 (±733.95)		2590.92 (±912.66)	
Dietary protein (% of total energy)				
HEI < 60	15.34 (±3.87)	0.96	17.94 (±4.64)	0.97
HEI ≥ 60	16.46 (±5.30)		17.39 (±4.28)	
Dietary carbohydrates (% of total energy)				
HEI < 60	50.32 (±7.62)	0.51	46.19 (±9.17)	0.3
HEI ≥ 60	51.55 (±9.58)		49.14 (±9.98)	
^†^ Dietary total fat (% of total energy)				
HEI < 60	34.99 (±5.91)	0.19	36.37 (±6.03)	0.7
HEI ≥ 60	32.48 (±7.10)		34.71 (±10.04)	
Dietary saturated fatty acids (% of total energy)				
HEI < 60	10.92 (±2.65)	0.16	12.50 (±2.88)	0.37
HEI ≥ 60	10.48 (±2.58)		11.27 (±2.86)	
Dietary monounsaturated fatty acids (% of total energy)				
HEI < 60	7.13 (±2.80)	0.76	8.23 (±2.91)	0.92
HEI ≥ 60	8.45 (±3.55)		8.70 (±4.12)	
Dietary polyunsaturated fatty acids (% of total energy)				
HEI < 60	4.34 (±2.25)	0.92	4.48 (±2.28)	0.98
HEI ≥ 60	4.74 (±2.37)		5.73 (±7.37)	
^†^ Dietary added sugars (% of total energy)				
HEI < 60	5.66 (±7.16)	0.69	3.22 (±3.05)	0.08
HEI ≥ 60	4.10 (±4.23)		4.83 (±3.82)	
% ^†^ AI total fiber				
HEI < 60	65.48 (±22.59)	0.05	69.45 (±44.28)	0.01
HEI ≥ 60	77.99 (±28.96)		88.60 (±28.58)	
% ^†^ AI sodium				
HEI < 60	304.57 (±108.58)	0.03	337.27 (±171.74)	0.22
HEI ≥ 60	242.70 (±102.50)		277.10 (±118.37)	
% RDA vitamin D				
HEI < 60	36.86 (±38.34)	0.49	38.64 (±34.69)	0.17
HEI ≥ 60	36.67 (±28.00)		50.61 (±35.32)	
% ^†^ RDA vitamin B_12_				
HEI < 60	197.52 (±141.82)	0.47	241.22 (±217.62)	0.04
HEI ≥ 60	215.40 (±124.05)		315.70 (±197.31)	
% RDA folate				
HEI < 60	52.14 (±17.15)	0.14	58.53 (±39.64)	0.12
HEI ≥ 60	68.21 (±44.26)		67.67 (±29.94)	
% RDA calcium				
HEI < 60	92.99 (±58.60)	0.13	112.34 (±70.01)	0.22
HEI ≥ 60	107.74 (±53.61)		130.36 (±63.31)	
% ^†^ RDA iron				
HEI < 60	56.34 (±20.04)	0.17	63.97 (±33.52)	0.08
HEI ≥ 60	74.22 (±49.91)		76.31 (±29.45)	
% ^†^ AI choline				
HEI < 60	69.22 (±44.44)	0.91	90.77 (±48.11)	0.04
HEI ≥ 60	68.82 (±40.68)		71.60 (±64.75)	
% ^†^ RDA zinc				
HEI < 60	81.23 (±46.81)	0.14	94.40 (±57.49)	0.04
HEI ≥ 60	96.24 (±51.76)		136.20 (±114.28)	

* Intake from dietary sources, excludes intake from supplements; ** *p*-values were obtained with Mann–Whitney U test, and were considered statistically significant if ≤0.05. ^†^ Differences between HEI categories were statistically significant; HEI—Healthy Eating Index; AI—adequate intake; RDA—recommended dietary allowance.

**Table 6:** Distribution of participants among HEI categories for surveys 1 and 2.

HEI Score Categories	Survey 1 (*n* = 51)		Survey 2 (*n* = 51)	
	Count	%	Count *	%
HEI ≥ 60	25	49.00%	17	33.30%
HEI < 60	26	51.00%	34	66.70%

HEI—Healthy Eating Index. * Differences between HEI categories were statistically significant based on the chi-squared test.

**Table 7:** Correlation coefficients (*r*) for HEI scores and the variables examined.

Variables	Survey 1 HEI Score	*p*-Value	Survey 2 HEI Score	*p*-Value
Knowledge of anemia score	0.41	0.003	0.32	0.024
Pre-pregnancy BMI	−0.31	0.03	0.05	0.73
Blood hemoglobin concentration	0.074	0.604	0.44	0.047

**Table 8:** Regression analysis predicting survey 1 HEI scores from maternal characteristics and nutritional knowledge.

	HEI						
Factors	B	SE B	β	t	Sig	R²	R² Changes
***Step 1***						0.119	0.119
Education	4.577	2.974	0.259	1.539	0.131		
Working status	1.732	1.156	0.232	1.498	0.141		
Marital status	0.225	2.601	0.016	0.086	0.931		
Age	0.16	0.366	0.075	0.437	0.664		
Ethnicity	−1.355	8.167	−0.026	−0.166	0.869		
***Step 2***						0.128	0.009
Education	4.99	3.43	0.282	1.45	0.155		
Working status	1.77	1.28	0.236	1.37	0.178		
Marital status	0.316	2.89	0.022	0.109	0.913		
Age	0.182	0.428	0.085	0.426	0.673		
Ethnicity	−1.26	8.69	−0.02	−0.146	0.885		
Smoking	−2.35	4.69	−0.09	−0.501	0.619		
Morning sickness	0.273	4.32	0.011	0.063	0.95		
Pre-pregnancy BMI	−0.721	2.33	−0.05	−0.308	0.759		
Food assistance programs	−0.094	4.36	−0.04	−0.021	0.983		
Blood hemoglobin concentration	0.037	6.85	0.001	0.005	0.996		
***Final step***						0.250 *	0.122 *
Education	3.686	3.273	0.208	1.126	0.267		
Working status	2.037	1.215	0.272	1.677	0.102		
Marital status	−0.057	2.72	−0.004	−0.021	0.983		
Age	0.194	0.402	0.09	0.481	0.633		
Ethnicity	−7.011	8.497	−0.135	−0.825	0.415		
Smoking	−2.772	4.414	−0.105	−0.628	0.534		
Morning sickness	−0.675	4.08	−0.026	−0.165	0.87		
Pre-pregnancy BMI	−0.209	2.207	−0.015	−0.095	0.925		
Food assistance programs	−0.117	4.103	−0.005	−0.028	0.977		
Blood hemoglobin concentration	−2.349	6.512	−0.062	−0.361	0.72		
Knowledge of anemia score	3.862	1.571	0.387	2.458	0.019 *		

HEI—Healthy Eating Index; BMI—Body Mass Index; * *p* < 0.05. Demographic data were entered in Step 1; food assistance programs, pre-pregnancy BMI, smoking, morning sickness, and blood hemoglobin concentration in Step 2; and knowledge of anemia in the final step.

**Table 9:** Regression analysis predicting survey 2 HEI scores from maternal characteristics and nutritional knowledge.

	HEI						
Factors	B	SE B	β	t	Sig	R²	R² Changes
***Step 1***						0.093	0.093
Education	−3.40	2.96	−0.195	−1.14	0.259		
Working status	1.03	1.15	0.14	0.894	0.377		
Marital status	2.73	2.59	0.192	1.05	0.298		
Age	0.263	0.365	0.124	0.719	0.476		
Ethnicity	1.17	8.15	0.023	0.144	0.886		
***Step 2***						0.271	0.117
Education	−4.28	3.09	−0.246	−1.38	0.174		
Working status	0.946	1.15	0.128	0.816	0.419		
Marital status	0.848	2.6	0.059	0.326	0.746		
Age	0.754	0.385	0.357	1.95	0.06		
Ethnicity	0.433	7.82	0.008	0.055	0.956		
Smoking	−3.40	4.25	−0.131	−0.806	0.425		
Morning sickness	−1.39	3.89	−0.055	−0.358	0.723		
Pre-pregnancy BMI	−0.735	2.1	−0.053	−0.349	0.729		
Food assistance programs	1.79	3.93	0.073	0.457	0.65		
Blood hemoglobin concentration	17.67	6.16	0.474 *	2.85	0.007 *		
***Final step***						0.350 *	0.08 *
Education	−5.31	2.99	−0.305	−1.77	0.084		
Working status	1.16	1.11	0.158	1.04	0.304		
Marital status	0.551	2.49	0.039	0.221	0.826		
Age	0.763	0.369	0.361	2.06	0.06		
Ethnicity	−4.12	7.78	−0.081	−0.530	0.599		
Smoking	−3.73	4.04	−0.143	−0.924	0.361		
Morning sickness	−2.14	3.73	−0.085	−0.574	0.57		
Pre-pregnancy BMI	−0.329	2.02	−0.024	−0.163	0.872		
Food assistance program	1.77	3.75	0.073	0.473	0.639		
Blood hemoglobin concentration	15.774	5.96	0.423	2.64	0.012 *		
Knowledge of anemia score	3.06	1.44	0.312	2.13	0.040 *		

HEI—Healthy Eating Index; BMI—Body Mass Index; * *p* < 0.05. Demographic data were entered in Step 1; food assistance programs, pre-pregnancy BMI, smoking, morning sickness, and blood hemoglobin concentration in Step 2; and knowledge of anemia in the final step.

## Data Availability

This is a research article, and all data generated or analyzed during this study are included in the published article. All inquiries should be directed to arezoo.rojhani@wmich.edu.
